# The role of income and frequency of dental visits in the relationship between dental sealant use and resin fillings after extended coverage: a retrospective cohort study

**DOI:** 10.1186/s12903-023-03387-z

**Published:** 2023-10-27

**Authors:** Dong-Hun Han, Hee-Yeon Kang, Jae-In Ryu

**Affiliations:** 1 Department of Preventive and Social Dentistry, Seoul National University School of Dentistry, Seoul, Republic of Korea; 2 Dental Research Institute, Seoul National University, Seoul, Republic of Korea; 3 Department of Health Policy and Management, Seoul National University College of Medicine, Seoul, Republic of Korea; 4 Department of Cancer Control and Population Health, National Cancer Center Graduate School of Cancer Science and Policy, Goyang, Republic of Korea; 5 Department of Preventive and Social Dentistry, Kyung Hee University College of Dentistry, Seoul, Republic of Korea

**Keywords:** Dental Health Services, Pit and fissure sealants, Dental Restoration, Socioeconomic factors, Dental Health Services

## Abstract

**Background:**

Prevention and treatment services use is closely associated with socioeconomic factors, such as income. This study aimed to investigate the relationship between implementing the sealant program and resin fillings restoration and to explore the role of income and frequency of dental visits in this relationship.

**Methods:**

This retrospective cohort study used the cohort database from the National Health Information Database of the National Health Insurance Service. The study population comprised 494,731 children born in 2007. A logistic regression model for the experience of resin fillings and a linear regression model for weighted utilization of them were used to identify the independent effects of dental sealants, income, and frequency of dental visits. All analyses were conducted using the SAS Enterprise Guide version 7.1 (SAS Institute Inc., Cary, NC, USA).

**Results:**

The ratio based on income level was almost proportional in all groups except the medical aid group, which had a rate as high as that of the wealthier group. Children without sealants were 1.05 times more likely to have resin fillings than others after adjusting for income level and frequency of visiting dental clinics in the final model. However, an opposite relationship between sealant experiences and resin fillings was observed in the previous model without dental visits. The gap in the weighted resin filling scores according to socioeconomic variables showed a similar tendency.

**Conclusions:**

Income and frequency of dental visits might be confounding factors for the relationship between dental sealant and resin fillings. It is necessary to consider the complex relationship between socioeconomic indicators and service use while studying oral health inequality.

## Background

Dental caries is a widespread disease that is the most prevalent condition and causes a burden of disease [1–3]. In addition, it can cause long-term damage [3]. Children with caries in their primary dentition have a very different caries trajectory in their permanent dentition compared with their caries-free contemporaries [4]. A high incidence of caries in adolescence is associated with socio-behavioral conditions, such as lower socioeconomic status or poor oral health-related behavior [5]. Many countries are trying to prevent tooth decay in the population, particularly in children. As a part of the efforts, they provide disease management of early childhood caries [6, 7], Childsmile [8–11], Teeth on Wheels [12], the UK FiCTION [13–15], and Northern Ireland Caries Prevention in Practice [16–19]. Some school-based programs have been implemented to deliver preventive dental services, School-Based Sealant Programs for children at high risk [20–22] and the Quebec Provincial Dental Public Health Program [23]. These programs achieved improved access to preventive dental care and decreased social inequity. Several governments provided dental services within national health coverage to ease the financial burden as well, such as National Health Services (NHS) or National Health Insurance (NHI) [24–26].

The provision of dental services influences access to and utilization of services according to the newly introduced conceptual framework of the “triangle of inequality” [27, 28]. Universal Health Coverage (UHC) as a provision dimension is key to increasing access to dental services and reducing the existing gap in using dental services [29]. However, several studies have reported the use of dental services could be limited by economic status even in the UHC. The ability to pay is closely associated with willingness to pay (WTP), which is defined as the maximum amount in monetary terms that an individual would be willing to sacrifice to obtain benefits [30, 31]. The WTP for dental prevention and treatment services was greater in the higher-income group than in the lower-income group [32]. Individuals with higher incomes even had a higher WTP for dental prevention, such as fluoride [33] than fillings [34]. Within this framework family conditions or socio-environmental factors were important determinants of inequality in affordability or availability of services.

Recently, the costs of dental sealants and light-cured resin fillings were covered by the National Health Insurance Service (NHIS) in Korea. Social insurance is one of the social security systems of Korea and the nation requires citizens to subscribe by law. As one of them, NHIS secures medical treatment and promotes health [35]. Coverage for dental sealants has been applied since 2009, and children aged under 19 years can be provided the service with 10% out-of-pocket payments, only for the molars. The use of dental sealants increased, and household payments decreased after inclusion in NHI [36]. Resin filling has been covered since 2019, with 30% out-of-pocket payment, permitted for premolars and molars of children under 13 years. After the inclusion of dental sealants within the NHI in 2009, the decline of untreated caries was greater in low-income households than in high-income households’ children [37]. However, others argued that the gap in oral health between socioeconomic status has not narrowed in adolescents, unlike children [38, 39]. Notably, these studies showed that there was limited effect on oral health status or reducing sealant use inequalities after coverage expansion. However, there is a lack of research exploring changes in the utilization of dental treatment services and their inequalities based on socioeconomic status after the provision of preventive services.

Therefore, this study aimed to determine the relationship between sealant use and resin fillings after the introduction of the sealant program and to explore the role of income and frequency of dental visits in this relationship.

## Methods

### Study design and data collection

This retrospective study used the National Health Information Database (NHID) of the NHIS [40–42]. As Korea’s health insurance system is mandatory for all citizens by law, all data on the use of medical institutions and pharmacies by people are stored and managed by the NHIS. The database is a large bank of data that includes 1.3 trillion cases of healthcare qualifications, including NHI or Medical Aid beneficiaries, insurance contributions, medical check-up results, treatment details, older adult long-term nursing insurance data, clinical status, and registered information on cancer and rare diseases, among others. The NHIS provides sample cohorts and customized databases through the National Health Insurance Sharing Service (NHISS) [43]. This retrospective cohort study used a database of Customized Health Information Data (NHIS-2021-1-444), which refers to the health information collected, managed, and maintained by the NHIS and is modifiable upon request for policy and academic research. We used a selected set of outpatient dental care data from NHID between 2014 and 2020. All diseases in the database were identified using the 10th Revision of the International Classification of Diseases (ICD-10). Following the Personal Information Protection Act, the organization provided data after the de-identification process. The analysis was conducted only inside the NHIS data analysis center, with a PC installed for data review and analysis. This study was reviewed and waived by the Institutional Review Board of Seoul National University School of Dentistry (S-D2210013).

### Study participants and variables

The study population was children born in 2007. Dental fillings with light-cured resin were selected as dependent variables for this study. There were three types of claim codes for light-curing composite resin fillings in the database: (1) one surface; (2) two surfaces, and (3) three surfaces and over. A weighted resin filling score was created to apply the treatment spectrum by multiplying the surface extent by the number of fillings. Since the detailed position of a tooth was unknown in NHID, only the overall experience of the dental treatments was used as a variable. The outcome variables were collected between 2019 and 2020. The independent variables were sociodemographic information, such as sex, disability, residential area, and income quintile associated with dental service utilization. People with disabilities in this study were defined as those who were officially registered with the Korean government as having a disability based on the Act on Welfare of Persons with Disabilities [44]. The Act defines a person with a disability as “a person whose daily life or social activity is substantially hampered by physical or mental disability over a long period”. The residential area was categorized as 17 districts in total, 8 metropolitan cities, and 9 provinces. It was classified into metropolitan and provincial areas. The income level was reclassified into quintiles using the 20th quantile variable of the insurance contributions. The medical aid program is a public assistance system in which the State guarantees medical problems of low-income citizens who are unable to sustain their lives and pays contributions for national health insurance due to earning under the minimum cost of living. The experience with dental sealants or frequency of visiting dental clinics during the last 5 years was also applied. The frequency of visiting dental clinics was divided into quartiles: 0) never; (1) 1^st^ (1–5 times); (2) 2^nd^ (6–10 times); (3) 3^rd^ (11–15 times); and (4) 4^th^ (16 times and over). It included any kind of visit for preventive, restorative, or oral examination from 2014 to 2018.

### Statistical analysis

This cohort data consisted of three types of Databases (DBs), including qualification, treatment, and clinic. It contains information on socioeconomic variables, the status of medical resource utilization, and the clinic. This study’s final dataset was created by merging DBs with an unidentifiable category of individuals. According to the independent variables, the utilization of dental fillings with resin and the weighted resin filling scores were examined using the chi-square and Kruskal–Wallis tests. A logistic regression model for the experience of dental fillings with resin and a regression model for the extent of them with weighted resin filling scores were used to identify the independent effects of dental sealant and socio-demographic information such as income. There was missing data only in income variables because in certain cases, insurance contribution is not charged to individuals, for example as professional soldiers, or it is charged afterward due to long-term leave. The descriptive analysis applied the total number of samples and regression analysis with the samples excluding missing data. All study samples were analyzed using the following models step-by-step: Model 1, adjusted for income quintile, experience or the number of dental sealants, and other variables such as sex or disability; Model 2, adjusted for Model 1 plus the frequency of visiting a dental clinic. Variance inflation factors (VIF) were used to assess multicollinearity among the socioeconomic variables. VIF > 10 indicated the presence of multicollinearity [45]. However, no indicators of multicollinearity were identified (VIF ≤ 10). The variables with p < 0.05 were identified to have significant effects on the dependent variable of the utilization of resin fillings. All analyses were conducted using the SAS Enterprise Guide version 7.1 (SAS Institute Inc., Cary, NC, USA).

## Results

A total of 494,731 children were included in this study (Table 1). There were more boys than girls, and medical aid beneficiaries were very few (approximately 2%). Approximately two-thirds of the students were in the fourth and fifth quintiles of household income and were more affluent and living in the city area. The number of students who had a disability was low (approximately 1%). One-third of the study sample cohort had received dental sealants during the last five years. Regarding dental fillings with light-cured resin, the rate reached approximately 30% of the cohort. All sociodemographic variables demonstrated statistically significant differences in the experience of dental fillings with resin (p < 0.001). Students who were girls, without disabilities, living in the metropolitan area, from wealthier families, had no experience with sealant, or visited dental clinics more often had a higher proportion of fillings. The proportion of people with resin fillings increased in all groups based on income level except the medical aid groups, which had a rate as high as that of the wealthier group. Finally, the utilization rate of resin fillings appeared as a U-shaped curve with a long tail for income. Similar patterns from the results of the weighted resin filling scores are shown in Table 2.

**Table 1:** The distribution of the study cohort at baseline and experience of resin filling between 2019 and 2020 in the National Health Insurance Database (NHID)

	Total		With resin filling (2019 ~ 2020)		
	N	(%)	N	(%)	
Total	494,731	(100.0)	148,347	(30.0)	
During last 5 years (2014 ~ 2018)					
Experience of sealant					
Yes	168,713	(34.1)	44,676	(26.5)	^***^
No	326,018	(65.9)	103,671	(31.8)	
Frequency of visiting dental clinic					
Never	15,909	( 3.2)	1,343	( 8.4)	^***^
1^st^ (1–5)	108,160	(21.9)	21,447	(19.8)	
2^nd^ (6–10)	146,223	(29.6)	40,473	(27.7)	
3^rd^ (11–15)	113,785	(23.0)	38,216	(33.6)	
4^th^ (≥ 16)	110,654	(22.4)	46,868	(42.4)	
In present (2019 ~ 2020)					
Sex					
Boys	254,455	(51.4)	69,985	(27.5)	^***^
Girls	240,276	(48.6)	78,362	(32.6)	
Disability					
Yes	5,269	( 1.1)	1,236	(23.5)	^***^
No	489,462	(98.9)	147,111	(30.1)	
Area of residence					
Province	150,340	(30.4)	39,486	(26.3)	^***^
Metropolitan	344,391	(69.6)	108,861	(31.6)	
Income quintile					
Medical aid	11,618	( 2.4)	3,282	(28.2)	^***^
1^st^ (poorest)	66,539	(13.7)	17,598	(26.4)	
2^nd^	42,510	( 8.8)	11,716	(27.6)	
3^rd^	65,835	(13.6)	17,997	(27.3)	
4^th^	115,504	(23.9)	35,024	(30.3)	
5^th^ (richest)	182,276	(37.6)	59,723	(32.8)	
missing	10,449				

^***^ p < 0.001

**Table 2:** The distribution of the study cohort with weighted resin filling score between 2019 and 2020

	Min	Max	Med	Mean	(SD)	
During last 5 years (2014 ~ 2018)						
Experience of sealant						
Yes	0	42	0	1.2	(2.8)	^***^
No	0	42	0	1.2	(2.6)	
Frequency of visiting dental clinic						
Never	0	27	0	0.4	(1.7)	^***^
1^st^ (1–5)	0	42	0	0.7	(2.1)	
2^nd^ (6–10)	0	34	0	1.0	(2.4)	
3^rd^ (11–15)	0	36	0	1.3	(2.7)	
4^th^ (≥ 16)	0	42	0	1.8	(3.2)	
In present (2019 ~ 2020)						
Sex						
Boys	0	42	0	1.1	(2.4)	^***^
Girls	0	38	0	1.4	(2.8)	
Disability						
Yes	0	32	0	1.1	(2.7)	^***^
No	0	42	0	1.2	(2.6)	
Area of residence						
Province	0	42	0	1.1	(2.6)	^***^
Metropolitan	0	40	0	1.2	(2.7)	
Income quintile						
Medical aid	0	42	0	1.3	(3.1)	^***^
1^st^ (poorest)	0	31	0	1.1	(2.6)	
2^nd^	0	36	0	1.1	(2.5)	
3^rd^	0	32	0	1.1	(2.6)	
4^th^	0	42	0	1.2	(2.6)	
5^th^ (richest)	0	38	0	1.3	(2.7)	

^***^ p < 0.001

A significant difference in having resin filling after adjusting for all sociodemographic information, such as income, experience with dental sealants, or frequency of visiting dental clinics during the last five years, is shown in Table 3 (all p < 0.001). The children without sealants were 1.05 times more likely to have resin fillings than those with sealants in Model 2. However, the opposite was observed in Model 1, demonstrating a higher probability of having resin fillings in the children with sealants. The experience of having resin fillings varied depending on whether the model included the dental visit frequency variable. In final Model 2 the difference in the experience of having resin fillings was higher with the frequency of dental clinic visits. Children who frequently visited the dentist were approximately 7.87 times more likely to receive resin filling. According to household income level, the children from the most affluent group were 1.22 times more likely to have resin fillings than those in the lowest income quintiles. The gap in the weighted resin filling scores of these variables in the multivariate linear regression models showed a similar pattern (all p < 0.001). There was a decreasing effect on the number of sealants and an increasing effect on the number of visits to dental clinics after full adjustment (Table 4).

**Table 3:** Odds ratio (OR) and 95% confidence interval (CI) estimated from logistic regression models for resin fillings

(= Reference)	Model 1	Model 2
During last 5 years (2014 ~ 2018)		
Sealants(= Yes)		
No	0.80(0.79–0.81) ^***^	1.05(1.03–1.06) ^***^
Frequency of visiting dental clinics(= Never)		
1^st^ (1–5)		2.67(2.51–2.83) ^***^
2^nd^ (6–10)		4.10(3.87–4.34) ^***^
3^rd^ (11–15)		5.40(5.09–5.73) ^***^
4^th^ (≥ 16)		7.87(7.42–8.34) ^***^
In present (2019 ~ 2020)		
Income quintile(= 1^st^ )		
Medical aid	1.12(1.07–1.17) ^***^	1.10(1.05–1.15) ^***^
2^nd^	1.05(1.02–1.08) ^**^	1.03(1.00-1.06) ^*^
3^rd^	1.04(1.01–1.06) ^**^	1.02(0.99–1.04)
4^th^	1.18(1.16–1.21) ^***^	1.13(1.11–1.16) ^***^
5^th^	1.30(1.27–1.32) ^***^	1.22(1.20–1.25) ^***^

^*^ p < 0.05, ^**^ p < 0.01, ^***^ p < 0.001. Model 1: adjusted for experience of dental sealants, sex, disability, area of residence, and income quintiles; Model 2: adjusted for Model 1 plus frequency of visiting dental clinic during last 5 years (categorical variable)

**Table 4:** Standardized correlation coefficient (B) and Standard Error (SE) estimated from multivariable linear regression models for weighted resin filling scores

(= Reference)	Model 1	Model 2
During last 5 years (2014 ~ 2018)		
No. of Sealant		
	0.04(0.00) ^***^	-0.09(0.00) ^***^
No. of visiting dental clinic during last 5 years		
		0.07(0.00) ^***^
In present (2019 ~ 2020)		
Income quintile(= 1^st^ )		
Medical aid	0.25(0.03) ^***^	0.21(0.03) ^***^
2^nd^	0.02(0.02)	-0.01(0.02)
3^rd^	0.02(0.01)	0.00(0.01)
4^th^	0.11(0.01) ^***^	0.06(0.01) ^***^
5^th^	0.18(0.01) ^***^	0.12(0.01) ^***^

^*^ p < 0.05, ^**^ p < 0.01, ^***^ p < 0.001. Model 1: adjusted for number of dental sealants, sex, disability, area of residence, and income quintiles; Model 2: adjusted for Model 1 plus frequency of visiting dental clinic during last 5 years (continuous variable)

## Discussion

This is the first analysis of the income-related effect on the relationship between dental sealants and resin fillings among Korean children aged 6 years using the NHID of the NHIS. This study’s results revealed that children without sealant were 1.05 times more likely to receive fillings with resin due to dental caries than those with sealant. The income gap was highest between the first and fifth quintiles of students, with students from rich families being 1.22 times more likely to have resin fillings.

The gap in resin treatment according to income level appeared as a U-shaped curve with a long tail. Among the children eligible for medical aid, 28.2% received resin treatment; however, the proportion of children in the lowest to the third income quintiles (26.4%, 27.6%, and 27.3%, respectively) was lower. Only children in the high-income quintiles (fourth and fifth quintiles) received resin treatment more than children eligible for medical aid (30.3% and 32.8%, respectively). This might happen because the out-of-pocket payment for resin treatment in NHI beneficiaries has been 30% since 2019. Paradoxically, this was supported by the higher use of services by the medical aid group, which had a relatively lower burden of user charges. Children aged under 18 years in medical aid could use the outpatient service with small out-of-pocket payments, of less than one dollar. For the people within NHI, the inverse care law was active, and people who most needed health care were least likely to receive it [46]. A previous study also reported that the higher-income group had a higher number of filled teeth with a reversed social gradient [47]. This deals with inequity in health care resulting in unfair social inequalities in the health care system [48] including dental care [49]. Poorer children with unhealthy oral conditions might need a higher amount of treatment if there are no obstacles to accessing dental services. In this case, the rate of children receiving resin fillings above the medical aid group could be higher than the affluent group. This study showed the opposite of expectations which means that there might be some difficulties in having dental treatment services for to who need it. On the other point of view, the data included in this study were collected in the early years of policy implementation. A study found socioeconomic gaps in untreated dental caries and sealants were alleviated several years later by the extended coverage for preventive services, especially in younger children aged under 12 years [38]. The inequality patterns of resin fillings according to income should be interpreted closely concerning untreated dental caries and sealants. Further monitoring and analysis of the relationships and patterns of dental status, preventive services, and dental treatments for dental caries are necessary to determine the true effects of policy implementation. If these differences continue even years after the universal coverage, the provision of service might be changed, including allocating caries prevention programs to target parents or children (school-based) directly [50] or providing the service not individually but school-based to increase access to care [51].

The difference in resin treatment according to the number of dental visits was very large. Children who visited the dental clinic the most frequently last five years were more likely to receive fillings with resin than those who had never visited the dental clinic (Table 1). First, the higher the number of dental visits, the higher the possibility of needing dental treatment, that is, the poor oral health condition. In this case, the possibility of resin fillings may increase in proportion to the number of dental visits. Alternatively, the relationship between the number of dental visits and resin fillings may have a positive, regardless of the oral health status. This may be supplier-induced demand or iatrogenic treatment under the payment system with fee-for-service [52–54]. However, it was impossible to determine the legitimacy of the treatment because the NHID provided only data on the dental service claimed not oral health status. Secondly, dental visits are guessed to be associated with socioeconomic conditions: the higher the household income or education level, the higher the possibility of dental visits [55–60]. Socioeconomic inequalities in dental visits start from an early stage of life and accumulate over time, thus inducing undesirable effects, especially in patients of low socioeconomic status [61]. Household income and education level had the greatest impact on the inequalities in filling treatments than caries experience as well [62]. Children in the present study who visited the dental clinic frequently in the past 5 years may have a higher household income level. Then the children from wealthier families have more chances to visit dental clinics and it resulted in a higher possibility of resin fillings.

There was a change in odds ratios of resin fillings for the children without sealant experiences. At first, the crude odds ratio was 1.29 compared to the children with sealant; the odds of children with resin filling were 0.466 and 0.360 according to the experiences of sealants, no and yes respectively (Table 1). Then, the odds ratio decreased to 0.80 after adjusting for sex, disability, residential area, and income (Model 1; Table 3). An opposite relationship was observed again after adjusting for Model 1 factors plus the frequency of dental visits (Model 2; Table 3). This explains the contradictory result of the logistic regression model adjusted for income without consideration of dental visits in Model 1. The frequency of dental visits was strongly associated with resin restoration. Therefore, the relationship between the past sealant experience and the current resin fillings may not be properly reflected if only the income level is considered. It can be said that the effect of the sealant factor appeared only after reflecting both the income level and the frequency of visits. The present study’s analysis revealed that the independent variable: sealant benefit, the dependent variable: resin restoration, and the covariate: dental visit, were influenced by socioeconomic conditions. Dental sealants applied to permanent molars’ occlusal surfaces effectively prevent caries in children and adolescents. Moderate-quality evidence showed that resin-based sealants reduced caries by 11–51% than no sealant when measured at 24 months follow-up. A similar benefit was observed at approximately 48 months of follow-up [63]. In addition to the preventive effect of sealants, there was also a systematic review of their economic evaluation. Comparing sealants with no sealants, the cost-effectiveness ratios in studies ranged from $41.96 per Decayed, Missing, and Filled Surfaces (DMFS) averted over 5 years or $45–$103 per quality-adjusted tooth years [64]. The present study revealed that the sealant reduced future resin restorations. This might mean preventive treatments such as dental sealants can help avoid additional dental costs among Korean children. The population of Korean children from low-income families at risk of dental caries is large. In 2018, 58.6% of the population aged 12 years had experienced dental caries, with those from high-income families accounting for 55.0% [65]. In contrast, children from low-income families demonstrated lower dental sealant prevalence than those from high-income families (53.7% vs. 61.1%). Therefore, increasing preventive dental treatments, such as dental sealants, could greatly impact reducing dental restorative treatments for dental caries.

This study had several strengths. First, this cohort study was based on a large cohort of Korean children. Second, the possible reverse causality was reduced by removing the source of bias such as the ‘temporality’ of the Bradford-Hill’s Causality Criteria [66–68] by selecting chronological order from preventive to treatment, such as sealant experience previously from 2014 to 2018, and resin restoration, in present from 2019 to 2020. Third, a long follow-up period was employed. Finally, careful adjustment of confounders, such as income level, residential area, and frequency of dental visits, was performed. However, this study also had several limitations: (1) lack of consideration for the incidence of dental caries and use of resin restoration as a surrogate index; (2) failure to consider other confounders such as oral health behaviors, sugar consumption, indirect cost, or travel time due to source of data; and (3) because the health insurance database was used, children who were outside of the NHI system could not be included in the analysis. Therefore, future longitudinal and compensated studies that will provide evidence for these aspects are needed.

## Conclusions

This cohort study provided further evidence regarding the preventive relationship between dental sealant use and future resin restoration due to dental caries considering income level and frequency of dental visits among children aged 12 years between 2019 and 2020. Because dental caries can cause pain, loss of function, reduced quality of life, and economic expenses, efforts to reduce dental caries should be encouraged. Future intervention studies are warranted to determine whether preventive oral healthcare is beneficial.

## Data Availability

The datasets generated during and analyzed during the current study are not publicly available due to the restriction applied by the National Health Insurance Service in the Republic of Korea but are available from the corresponding author on reasonable request.

## List of abbreviations

NHS: National Health Services
NHI: National Health Insurance
UHC: Universal health coverage
WTP: Willingness to pay
NHIS: National Health Insurance Service
NHID: National Health Information Database
VIF: Variance inflation factors
